# Metagenome-assembled genome reveals species and functional composition of Jianghan chicken gut microbiota and isolation of *Pediococcus acidilactic* with probiotic properties

**DOI:** 10.1186/s40168-023-01745-1

**Published:** 2024-02-12

**Authors:** Hongye Shen, Tinghui Wang, Weiwei Dong, Guoping Sun, Jun Liu, Nan Peng, Shumiao Zhao

**Affiliations:** 1 National Key Laboratory of Agricultural Microbiology and College of Life Science and Technology, Huazhong Agricultural University, Wuhan, 430070 China; 2 Hubei Poder Biotechnology Co., Ltd, Huangshi, 435000 China; 3 College of Life Sciences, Hubei Normal University, Huangshi, 435000 China

**Keywords:** Free-range chicken, Gut microbiota, Metagenomic sequencing, Potential probiotics, Antibiotic resistance genes

## Abstract

**Background:**

Chickens are one of the most widely farmed animals worldwide and play a crucial role in meat and egg production. Gut microbiota is essential for chickens’ health, disease, growth, and egg production. However, native chickens such as Jianghan chickens have better meat and egg production quality than centralized chickens, their intestinal microbial diversity is richer, and the potential gut microbial resources may bring health benefits to the host.

**Results:**

The bacterial species composition in the gut microbiota of Jianghan chickens is similar to that of other chicken breeds, with Phocaeicola and Bacteroides being the most abundant bacterial genera. The LEfSe analysis revealed significant differences in species composition and functional profiles between samples from Jingzhou and the other three groups. Functional annotation indicated that the gut microbiota of Jianghan chickens were dominated by metabolic genes, with the highest number of genes related to carbohydrate metabolism. Several antibiotic resistance genes (ARGs) were found, and the composition of ARGs was similar to that of factory-farmed chickens, suggesting that antibiotics were widely present in the gut microbiota of Jianghan chickens. The resistance genes of Jianghan chickens are mainly carried by microorganisms of the Bacteroidota and Bacillota phylum. In addition, more than 829 isolates were selected from the microbiota of Jianghan chickens. Following three rounds of acid and bile tolerance experiments performed on all the isolated strains, it was determined that six strains of *Pediococcus acidilactici* exhibited consistent tolerance. Further experiments confirmed that three of these strains (A4, B9, and C2) held substantial probiotic potential, with *P. acidilactici* B9 displaying the highest probiotic potential.

**Conclusions:**

This study elucidates the composition of the intestinal microbiota and functional gene repertoire in Jianghan chickens. Despite the absence of antibiotic supplementation, the intestinal microbial community of Jianghan chickens still demonstrates a profile of antibiotic resistance genes similar to that of intensively reared chickens, suggesting resistance genes are prevalent in free-ranging poultry. Moreover, Jianghan and intensively reared chickens host major resistance genes differently, an aspect seldom explored between free-range and pastured chickens. Furthermore, among the 829 isolates, three strains of *P. acidilatici* exhibited strong probiotic potential. These findings provide insights into the unique gut microbiota of Jianghan chickens and highlight potential probiotic strains offering benefits to the host.

Video Abstract

**Supplementary Information:**

The online version contains supplementary material available at 10.1186/s40168-023-01745-1.

## Introduction

Chickens are one of the most widely used farm animals worldwide and are an important source of meat and eggs. It is estimated that over 60 billion chickens are produced worldwide and over 12 million tons of chicken is produced annually [1]. The gut microbiome is a complex and diverse ecosystem with numerous metabolic and immune functions that is crucial to the health and productivity of chickens [2]. The chicken gut microbiota is highly diverse, with many microorganisms inhabiting the gastrointestinal tract. These microorganisms provide hydrolases for the animal and convert carbohydrates into energy through fiber fermentation [3, 4], among other functions. Previous studies have indicated that the chicken intestinal microbiome plays a vital role in nutrient degradation [5], immune system development [6], pathogen elimination [7], abdominal fat mass [8], and feed efficiency [9]. The diversity of intestinal microbiota plays a crucial role in influencing the host’s health, production performance, and disease susceptibility [10–12]. Understanding the role of the chicken gut microbiome is critical to manipulating the gut microbiome to promote chicken health and improve productivity. At present, the intestinal flora of poultry can be adjusted by feeding lactic acid bacteria to inhibit intestinal bacterial infection, or the composition of intestinal microorganisms can be affected by adding plant feed (tea, leaves, allium hookeri, etc.) to improve the health of chickens. Directly use prebiotics to improve the intestinal microbiome of chickens and improve their immunity [13–17]. However, before improving the gut microbiota of chickens, it is essential to have a comprehensive understanding of the gut microbiota. Existing studies have established chicken gut microbiome metagenomic datasets and unveiled the impact of certain antibiotics on chicken growth, and extensive microbial diversity within the chicken gut microbiome revealed by metagenomics and culture [18, 19]. In addition, there are significant variations in the composition of the chicken intestinal microbiota based on region, age group, sex, and feeding method, which have notable implications for both chicken production and health [20]. However, existing studies have insufficiently understood differences in the composition and function of gut microbiota in chickens under different feeding methods (free range, intensive rearing), which is very important. Studies have shown that the meat quality of free-range chickens is better than that of centralized chickens [21], but the potential contribution of gut microbes has not been revealed. Currently, there is a lack of research on the species composition, functional composition, and screening of potential probiotics in the gut microbial community of free-range chickens.

In recent years, high-throughput sequencing technology has emerged as a powerful tool for investigating the species and functional composition of microbial communities [22]. This technology greatly facilitates the exploration of the functionality of chicken gut microbiota. Presently, a detailed examination of the gut microbiomes in poultry and broiler chickens has been performed using amplicon sequencing technology to reveal the bacterial diversity present within these environments [23, 24]. However, 16S rRNA amplicon sequencing may limit our understanding of microbial function because it can only measure specific gene fragments in a sample [25]. Our previous studies of chicken gut microbiota have also been constrained by their reliance on either amplicon sequencing techniques, but understanding of the composition and function of gut microbiota in chickens remains limited. The advent of high-throughput sequencing and metagenomic sorting technologies has made it possible to obtain nearly complete metagenomic assembled genomes (MAGs) on a large scale [26]. This technology has generated thousands of MAGs from chicken cecal microbes, making metagenomic sequencing a reliable and effective method for investigating the chicken gut microbiota [27].

As research on the gut microbiota continues to advance, we increasingly recognize the significant contributions of the gut microbiota to the health and growth of poultry. Jianghan Chicken is an excellent local breed of chicken on the Jianghan Plain in China, and it is widely farmed in rural areas of central China. Jianghan chicken is cherished among the Chinese populace for its delectable meat and superior egg quality; however, there is a notable scarcity of research regarding the gut microbiota of Jianghan chickens. Therefore, we hypothesized the following: (1) The microbial species composition and functional profile of the gut microbiota of Jianghan chickens differ from those of industrially raised meat and egg chickens, potentially contributing to their superior edibility. (2) Jianghan chickens’ gut microbiota may harbor undiscovered probiotic strains, which confer numerous benefits to the host. Thus, this study aimed to comprehensively and accurately investigate the gut microbes and functional composition in Jianghan chickens. Our research focused on the gut microbiota of 34 Jianghan chickens from four cities in the Hubei province of the Yangtze River Basin using metagenomic sequencing technology. A total of 829 strains of chicken gut microbiota were successfully identified, and microorganisms with comprehensive probiotic capabilities were isolated. This study provides a list of the MAG of Jianghan chicken microbiome, elucidates the intestinal microbial characteristics. Furthermore, it offers a valuable microbial resource for further investigation into the gut microbiota of indigenous chickens. In summary, this research provides insights into the gut microbiota of Jianghan chickens, underscoring the importance of the microbial ecosystem in maintaining their health and productivity.

## Results

### Community richness and diversity of microbiota in the chicken gut from different regions

**Fig. 1:**
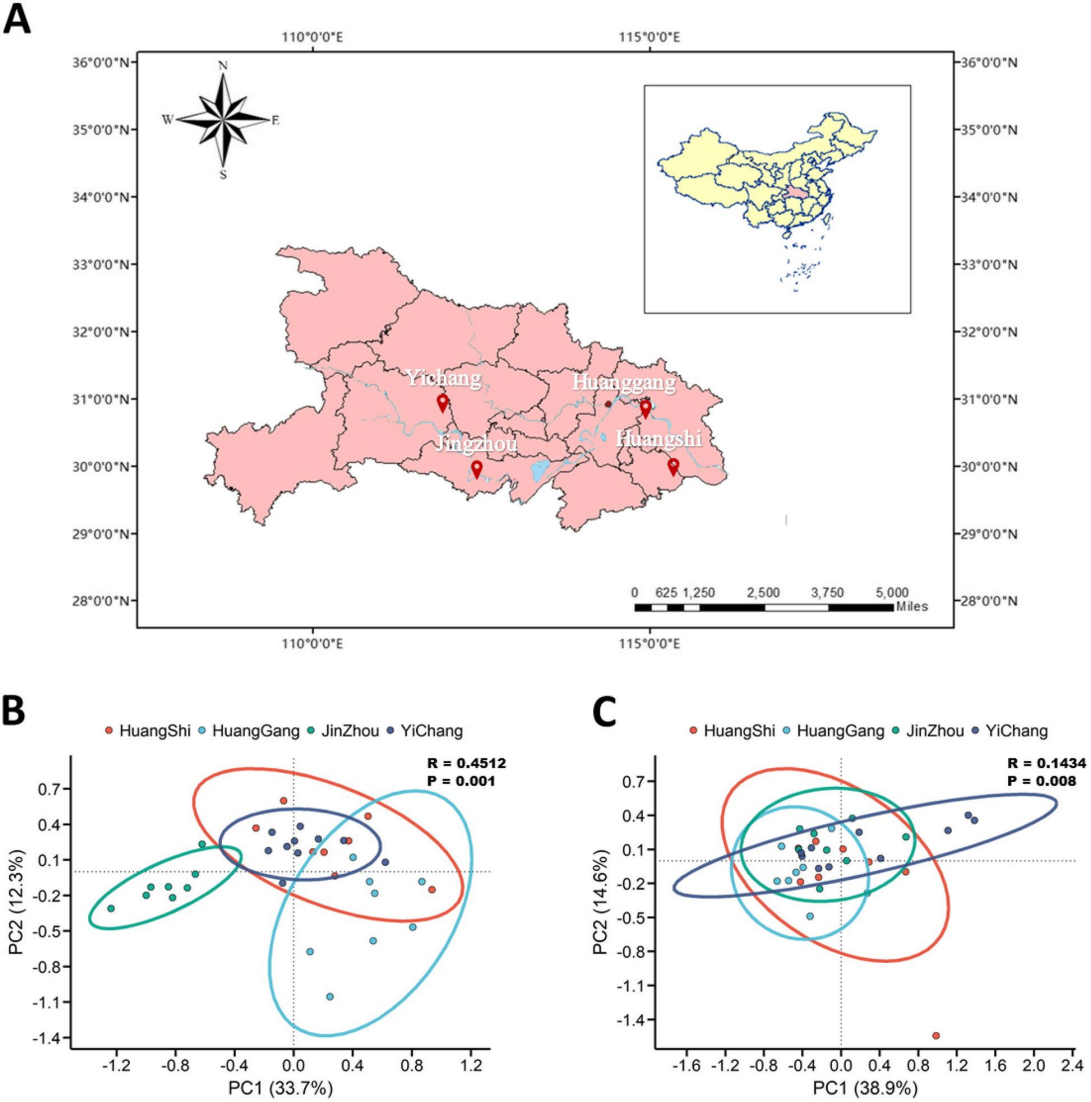
Geographically dependent characteristics of the composition of the chicken gut microbiota. **A** Distribution of the four sampling sites (Yichang, YC; Jingzhou, JZ; Huangshi, HS; Huanggang, HG) in China. All four sampling sites are located near the Yangtze River basin within Hubei Province. **B** Principal component analysis plot showing the composition of bacterial microbiota among 34 samples of YC, JZ, HS, and HG. **C** Principal component analysis plot showing the composition of fungal microbiota among 34 samples of YC, JZ, HS, and HG

In our research, a total of 400 Gb of Illumina sequencing data were obtained from the 34 collected samples, and after quality control, 2.7 billion high-quality reads were obtained with an average sequence length of 142.84 bp. To investigate the variation in the composition of microbial communities in the gut of free-range chickens across different regions of Hubei Province, we performed a principal component analysis (PCA) based on the top thirty genera in terms of average abundance, as annotated by bacterial and fungal species. For the bacterial communities (Fig. 1B), the JZ sample showed a significant difference compared to the other regional samples, while the HS and YG samples exhibited partial similarity. The Analysis of Similarities (ANOSIM) test for the bacterial microbiota yielded an R = 0.4512 and *P* < 0.001, indicating a significant difference between the microbiota samples from the four regions. Regarding the fungal community (Fig. 1C), there was a large overlap among the four regions, with only a few sample points showing significant deviation between YC and HS. After ANOSIM test, the R = 0.1434 and *P* = 0.008 indicated that the microbial community samples from the four different regions were significantly different from each other. However, the degree of variation in the fungal community was not as high as that in the bacterial community. Overall, the composition of bacterial communities in the four samples showed significant difference, with the JZ sample being significantly different from the other three groups. In contrast, the composition of fungal communities showed a similar pattern across the four regions.

### Microbial community abundance in the four regions

**Fig. 2:**
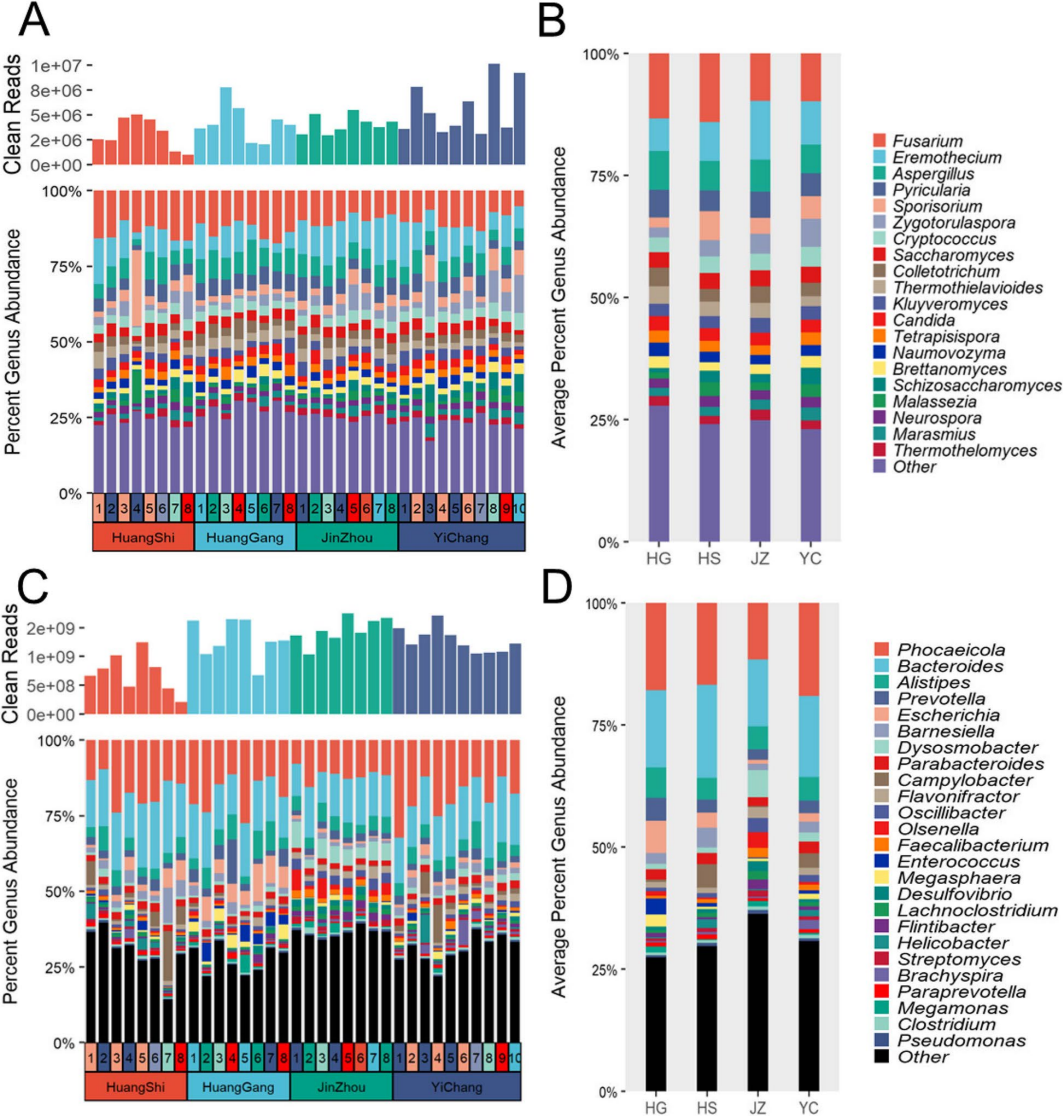
Composition and dynamic of microbial community at the genus level (fungal top 20, bacterial top 25), the numbers represent repeated samples in each region. **A** The dynamic of fungal community at four sites. **B** The composition of fungal community based on the average of every site. **C** The dynamic of bacterial community at four sites. **D** The composition of microbial community is based on the average of every site (*, *P* ≤ 0.05; **, *P* ≤ 0.01; ***, *P* ≤ 0.001)

In this study, we performed metagenomic sequencing of chicken gut microbiota from four different regions and identified 1698 bacterial and 42 fungal microbial genera. Among them, the top 25 bacterial and 20 fungal genera in terms of abundance were selected for analysis. As the majority of the sequences were annotated as bacteria, this study focused on the relative abundance of the chicken gut bacterial communities across the four regions. At the bacterial genus level (Fig. 2C), *Phocaeicola* (16.3%), *Bacteroides* (16.2%), *Alistipes* (5.03%), *Prevotella* (3.03%), and *Parabacteroides* (2.15%) were identified as abundant bacterial species, with relative abundance > 1% at all sampling sites [28]. The bacterial microbial composition of samples from HG, HS, and JZ regions was similar (Fig. 2D), with no significant differences observed in the mean relative abundance of the five abundant microbial species, except for Bacteroides (*P* = 0.04).

Only 42 fungal sequences were identified in the sequencing data of the chicken gut microbiota, indicating a lower abundance of fungi than bacteria. As shown in Fig. 2A, *Fusarium* (11.72%), *Eremothecium* (8.86%), *Aspergillus* (6.62%), *Pyricularia* (4.97%), *Sporisorium* (3.96%), *Cryptococcus* (3.52%), *Saccharomyces* (3.23%), *Thermothielavioides* (2.91%), *Candida* (2.70%), *Tetrapisispora* (2.31%), *Naumovozyma* (2.26%), and *Brettanomyces* (2.16%) were identified as abundant fungal species, with relative abundance above 1% at all sampling site [28]. From the group mean 15 genera in the fungal community ranged from 2 to 3.8% (Fig. 2B), suggesting a relatively homogeneous distribution of fungal community species. No significant differences were observed in the composition of the fungal microbial community across the four regions.

### Functional annotation using COG, KEGG, and CAZy databases

**Fig. 3:**
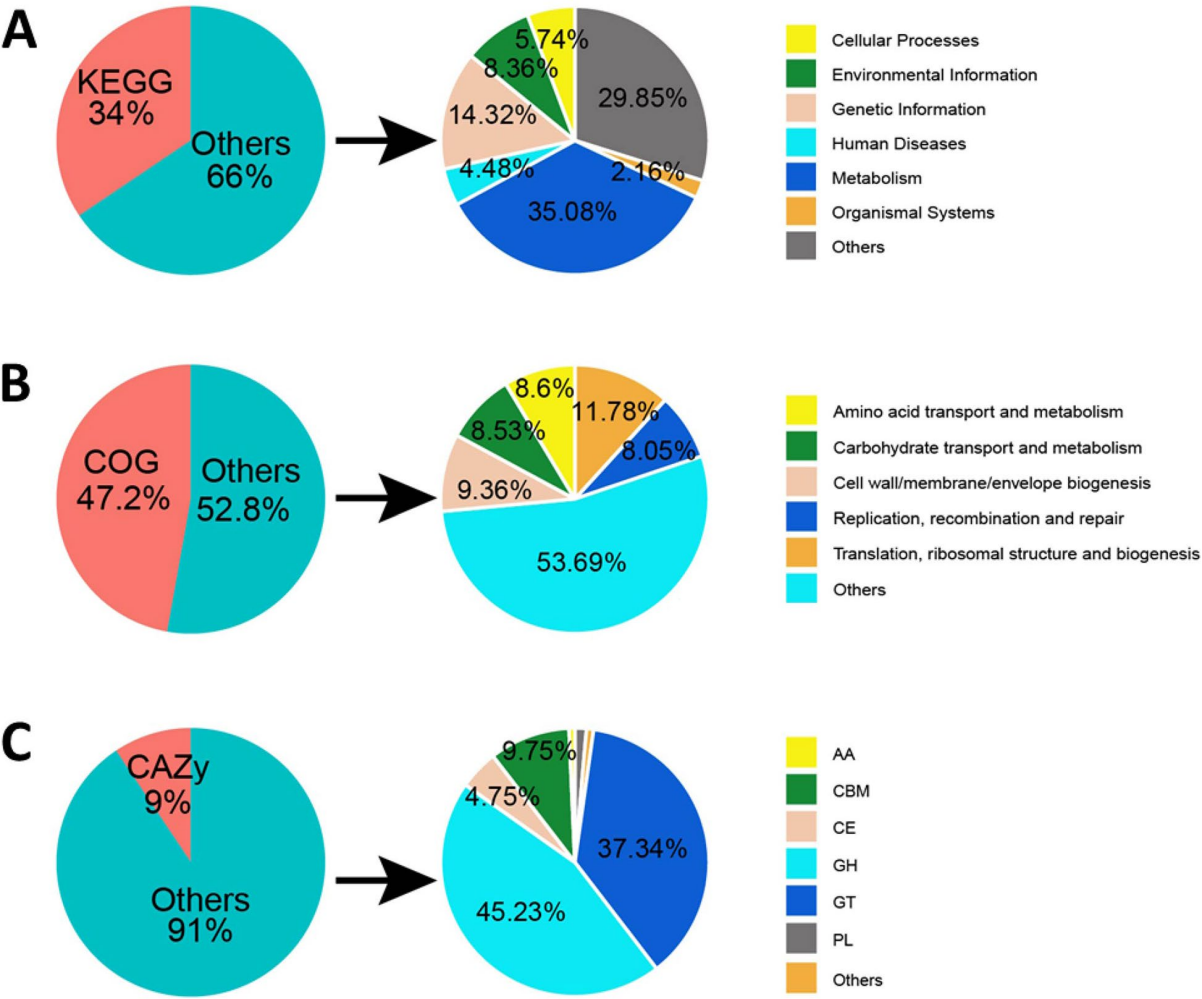
Functional annotation of MAG in the chicken gut. Functional annotations of chicken microbial proteins. Annotation results were obtained using KEGG (**A**), COG (**B**), and CAZy (**C**)

To functionally annotate each non-redundant protein sequence, we utilized several databases including COG [29], KEGG [30], GO [31], and CAZy [32]. Of the total non-redundant protein sequences, 34.44%, 47.20%, and 31.45% were annotated in the KEGG, COG, and GO databases, respectively. Our analysis showed that 34%, 47.2%, and 9% of the predicted proteins had at least one KEGG, COG, and CAZy function, respectively (Fig. 3).

The KEGG annotation results are shown in Fig. 3A. We observed that the protein functions were classified into six metabolic systems, with the highest proportion of proteins playing metabolic functions, accounting for approximately 35.08% of total proteins. Among all the metabolism-related genes, those related to carbohydrate and amino acid metabolism were predominant. These results are similar to those obtained from the COG database, indicating that the functions of the chicken gut microbes were mainly related to metabolism and that the carbohydrate and amino acid metabolic pathways were the most dominant metabolic pathways. This observation is attributed to the daily diet of the chickens, which is mainly based on cereals, insects, and vegetable leaves.

Figure 3B shows the annotated results in COG, where we found that the most abundant gene sequences were responsible for the translation, and production of ribosomal structures, followed by functional genes for the biogenesis of cell wall/membrane/envelope. The high abundance of these proteins indicated that the chicken gut microbial community was metabolically active and capable of constant metabolism and division to produce offspring. Moreover, proteins related to sugar metabolism, amino acid transport and metabolism, and lipid transport and metabolism suggest that the chicken gut microbial community has a strong metabolic potential for various nutrients and a high capacity for division and metabolism.

After identifying the carbohydrate metabolism-associated proteins as the predominant metabolic genes, we annotated the data using the CAZy database (Fig. 3C). Our analysis revealed that 9.4% of the genes were annotated and glycoside hydrolases (GHs) and glycosyl transferases (GTs) were the most abundant functional proteins involved in carbohydrate metabolism, accounting for 45.23 and 37.34% of the total fractionation, respectively. The carbohydrate-binding protein modules (CBMs) and carbohydrate esterases (CEs) were also relatively abundant with a fraction of approximately 14.5%, and these two functional genes can promote carbohydrate metabolism. The relative abundance of auxiliary activities (AAs) and polysaccharide lyases (PLs), which act on polysaccharide catabolism, was lower at 2%. The annotated results of the CAZy database suggested that the carbohydrate metabolism genes of the chicken gut microbial community were primarily involved in the synthesis of GHs and GTs, and some genes functioned to provide CBMs and assist in the catabolism of PLs. Our findings provide insight into the carbohydrate metabolism in the chicken gut microbial community and offer a promising source of enzymes and microbes for fermentation biotechnology industries [33, 34].

### Functional annotation using KEGG, CARD, and CAZy from different locations

**Fig. 4:**
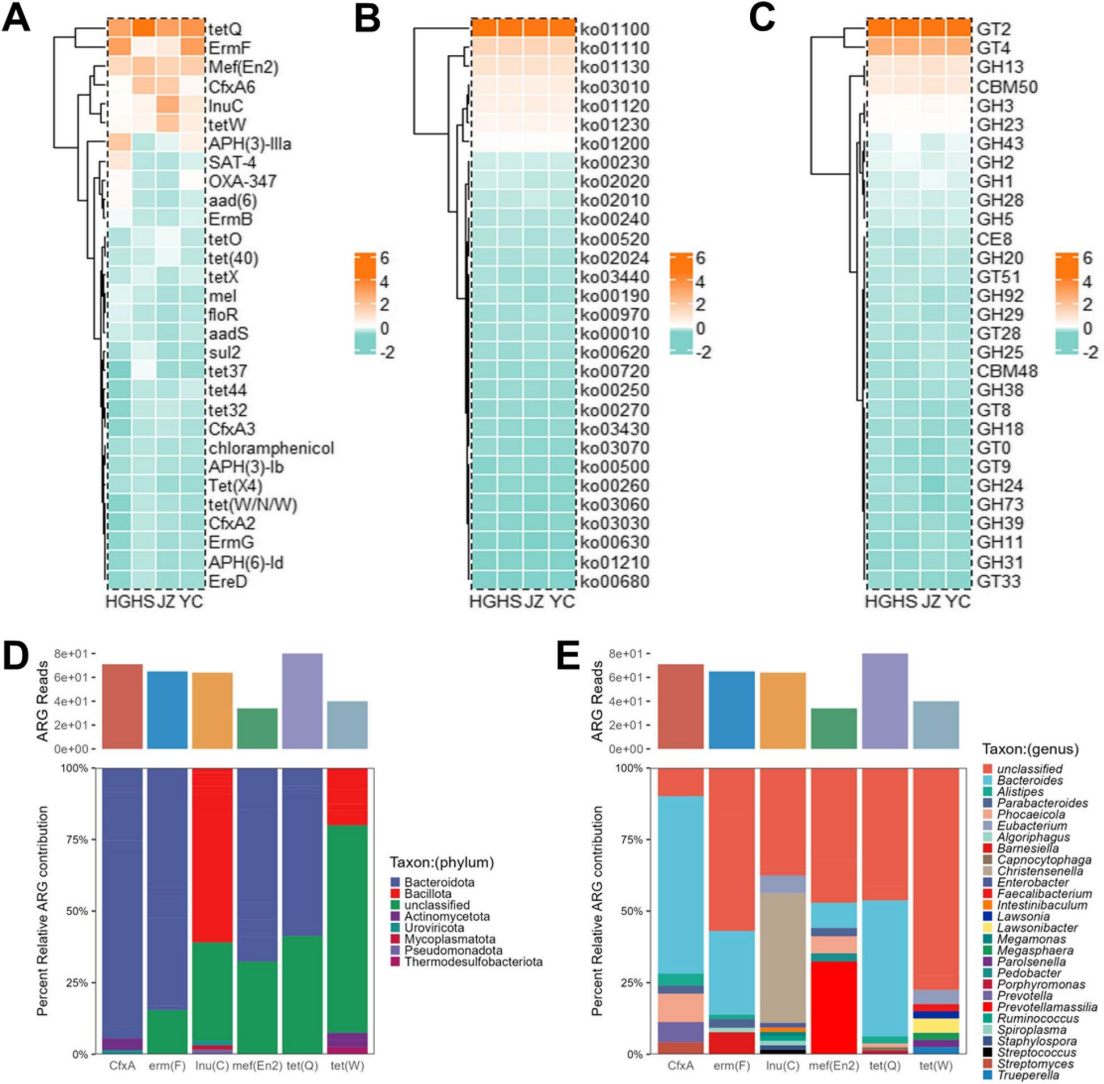
Clustering heatmap of functional gene distribution and relative abundances for samples collected from different site based on the three types of databases. **A** CARD database, **B** KEGG database, **C** CAZy database. Only relative abundances of top 30 genes were considered to the dominant gene

Antibiotics are crucial for controlling harmful microorganisms. However, abuse and misuse of antibiotics can lead to the development of antibiotic tolerance or resistance in microorganisms via horizontal gene transfer or genetic variation. To explore the composition and differences in resistance genes among chicken gut microbes, we used the CARD database to predict the antibiotic resistance genes in the gut. The standardized annotated results for antibiotic resistance genes (ARGs) are displayed as a heatmap (Fig. 4A), revealing variation in the number of ARG types in the four regional samples. The highest number of ARGs was found in the HG and HS samples. Among all the resistance genes, the tet family (tetW, tetO, tet (40), tetX, tet37, tet44, tet32, and tet (W/N/W)) resistance genes, primarily tetQ, had the most abundant tetracycline resistance genes. Additionally, streptavidin-resistant genes (ErmF), macrolide antibiotic resistance genes (Mef (en2)), and cephalexin-resistant genes (CfxA6) were the five most abundant resistance genes, collectively accounting for over 60% of the overall number. Based on the annotation of resistance genes, tetracyclines, streptogramins, lactones, and cephalosporins were the main types of resistance genes in chickens from the four regions. The host-tracking analysis identified Bacteroidota and Bacillota as the major ARGs hosts at the phylum level (Fig. 4D). At the genus level, the main ARGs hosts are *Bacteroides* and *Alistipes* (Fig. 4E).

The KEGG database was used to predict metabolic pathways (Fig. 4B). The results of the KEGG database annotation for the four groups of samples were highly similar in terms of the top 30 functional pathways, with metabolic function being the most dominant pathway. Finally, annotation results in the CAZy database revealed that GHs and GTs were the predominant carbohydrate-active enzymes (Fig. 4C), accounting for 27 of the top 30 most abundant enzymes. In addition, a small number of CBMs and a CE8 were also present in similar composition and abundance across the four regions.

### Distribution and variation of major microorganisms in different regions

**Fig. 5:**
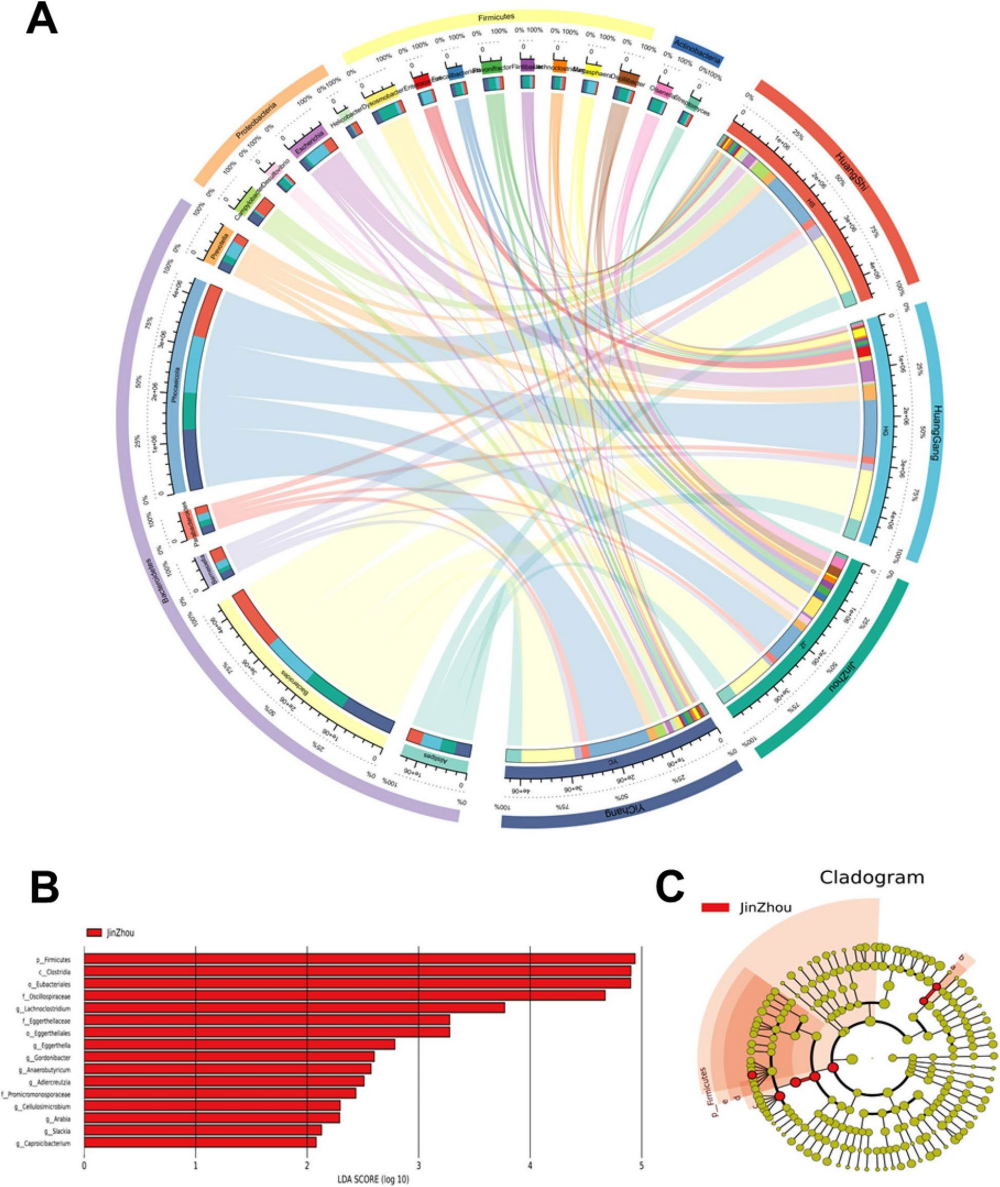
**A** Distribution of the main genus-level microorganisms in samples from four sites. The half semicircles on the left show the percentage of major microorganisms (top 20), the genus-level and the phylum-level annotations. The right semicircle shows the composition and reads of the main microorganisms (top 20) based on the average of every site. LEfSe analysis of microbial abundance between the four samples. **B** is the LDA score. **C** is a cladogram of the microbial community

Most of the microbial taxa identified in the chicken gut samples were classified as bacteria. To investigate the regional differences in microbial community composition and sequencing abundance, we analyzed the average abundance of bacterial taxa across the four regional samples, selecting the top 20 genera for the construction of a heatmap. As shown in Fig. 5A, there was minimal divergence in bacterial community structure across the four regions, with the most abundant phylum being Bacteroidetes, followed by Firmicutes, Proteobacteria, and Actinobacteria. For highly abundant genera, such as *Phocaeicola*, *Bacteroides*, and *Alistipes*, the distribution across regions was similar and not significantly different, whereas for low-abundance genera, some variation was observed.

Further analysis of the low-abundance microbial taxa identified regional differences in community structure. LEfSe analysis was conducted to identify the differentially abundant microbial genera among the four regions. As shown in Fig. 5B and C, all significant biomarkers were associated with samples from JZ and were primarily members of the phylum Firmicutes, class Clostridia, order Eubacteriales, family Lachnospiraceae, and various microbial genera within the families Oscillospiraceae and Eggerthellales. Alpha diversity analysis revealed that microbial diversity in JZ samples was significantly higher than that in samples from other regions, and PCA revealed distinct differences in bacterial community structure in JZ samples relative to those from other regions. Collectively, these analyses support the conclusion that the microbial community structure of the JZ samples differed from that of the other three regions.

### Distribution and variation of functional proteins in different regions

**Fig. 6:**
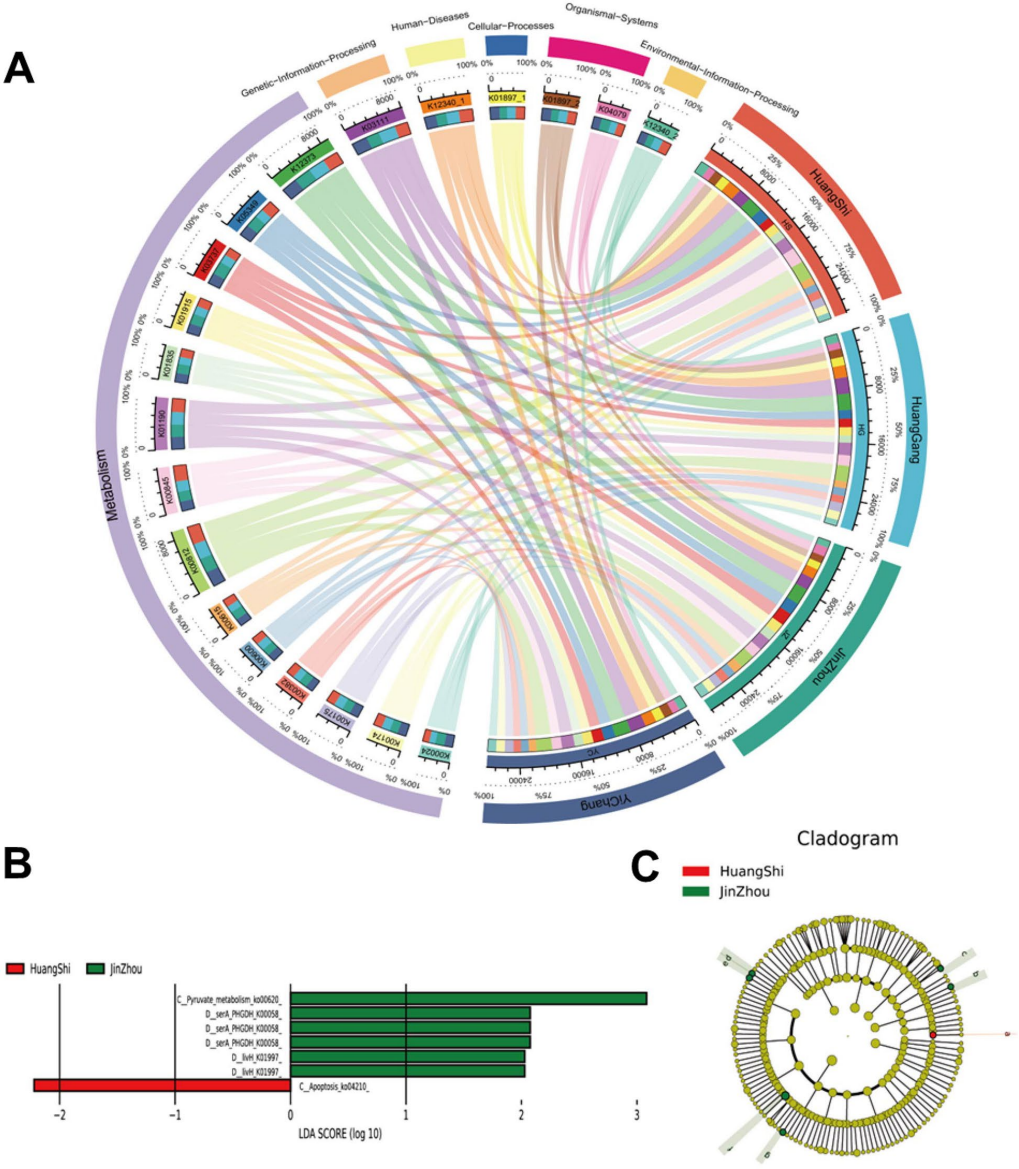
**A** Distribution of the main functional genes in samples from four sites. The left semicircles show the percentage of major functional genes (top 20), the level 1 and the level 4. The right semicircles show the composition and reads of the main functional genes (top 20) based on the average of every site. LEfSe analysis of functional genes abundance between the four samples. **B** is the LDA score. **C** is a cladogram of the functional genes

In the present study, we performed sequence annotation using multiple databases to comprehensively understand the functional capacity of chicken gut microbiota. To investigate the metabolic functions of chicken gut microbes, we utilized the KEGG database to predict metabolic pathways. In addition, we analyzed the functional annotation results from the four regional samples by selecting the top 20 functional genes with the highest abundance for string plots. Figure 6A illustrates the abundance levels of the 20 most prevalent enzyme genes, which were evenly distributed across the sites. Metabolic functions accounted for > 70% of the total enzyme genes, with carbohydrate metabolism being the most abundant.

To identify the potential differences in functional composition across regions, we employed LEfSE analysis to identify marker enzymes. The analysis revealed that most all marker enzymes were from JZ, with only one signature gene sequence from another region (Fig. 6B and C). The differential gene pathways from JZ were K00620, K00058, and K01997, of which K00058 expressed D-3-phosphoglycerate dehydrogenase or 2-oxoglutarate reductase, which are involved in various metabolic processes. K01997 expresses branched-chain amino acid transport system permease protein, which plays a role in the processing of cellular and environmental information. Our findings suggest that there may be regional differences in the functional composition of chicken gut microbes, with JZ having a distinct functional profile compared to the other regions.

Overall, our results provide insights into the functional capacity of chicken gut microbiota and highlight the potential regional differences in their metabolic functions. These findings may have implications for the development of novel strategies to improve for poultry health and productivity.

### Isolation of potential probiotics from chicken gut

Intestinal microorganisms are crucial for the production of probiotics and immune-enhancing microorganisms [35]. Lactic acid bacteria are among the most extensively studied microorganisms and have been utilized in various fields, including numerous applications related to the enhancement of gut microbiota [15]. Through previous metagenomic analysis, we found that the gut microbes of Jianghan chickens are rich in microbial resources and have strong metabolic capabilities. The analysis showed that the samples from Jingzhou had unique microbial community and metabolic function composition. Lactic acid-producing bacteria is most used probiotic genera [36], the average abundance of *Lachnoclostridium* in JZ samples was 1.73%, while that in the other three regions was 0.8 to 0.9%. Therefore, for the isolation and screening of bacteria from the four groups of samples, we focused on the isolation of the JZ samples. Metagenomic sequencing was employed to isolate and identify the gut microbiota of Jianghan chickens. The composition and diversity of the intestinal microbiota in each region were determined using a combination of metagenomic analysis, culture, and sequencing.

**Fig. 7:**
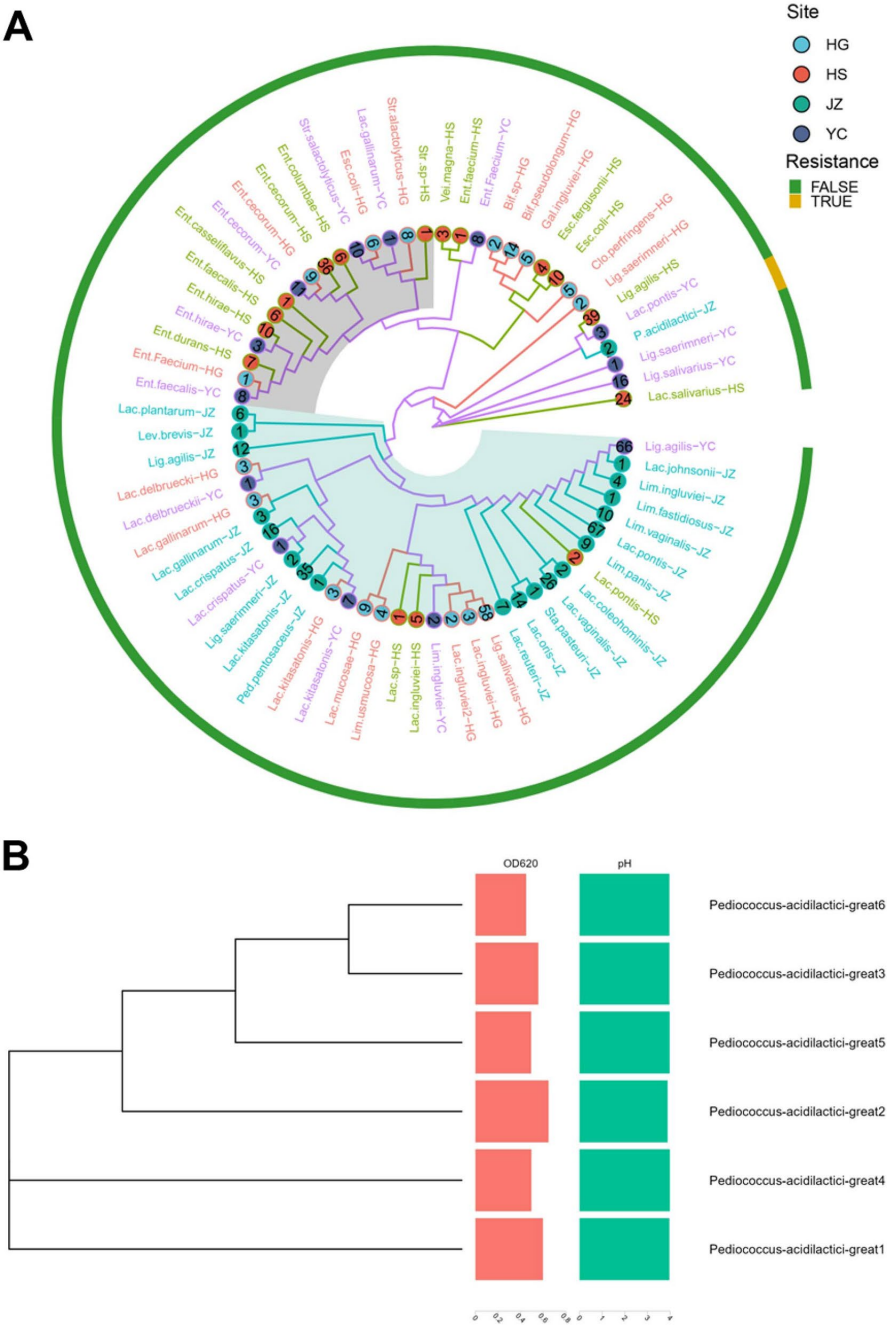
**A** Phylogenetic tree of 829 isolated chicken intestinal microorganisms. The inner circles depict taxonomic assignments for the microorganisms, the colors of the branches and nodes represent different sampling sites. The taxa names are labeled, and the number of different taxa within each species is provided at the nodes. The color of the outer ring represents resistance to bile salts, with TRUE representing resistance and FALSE representing no resistance. **B** Six phylogenetic trees with bile salt-resistant strains; the bar-plot shows the absorbance and pH of the bacterial solution after 24 h growth

The appropriate medium and screening method was selected for large-scale cultivation and identification of the chicken gut microbiota. The caeca of chickens were collected, and anaerobic and aerobic cultures were conducted on multiple media. Eight hundred twenty-nine strains were isolated, with the majority being identified as lactic acid-producing bacteria using 16S rRNA sequencing. The tolerance and acid-producing ability of the isolated strains were evaluated, and most strains could not grow in a medium supplemented with 0.2% bile salt. After multiple rounds of screening, six strains of *P. acidilactici* isolated from the JZ samples were identified as having stable bile salt tolerance. A phylogenetic tree was constructed using the optimal tree-building model to demonstrate the kinship among the six strains (Fig. 7B). The concentration of these six strains, as represented by the OD_620_ value, reached 0.5 after 24 h of growth in the culture medium with 0.2% bile salt, while the pH value ranged between 3.7 and 3.8 after 24 h of growth.

**Fig. 8:**
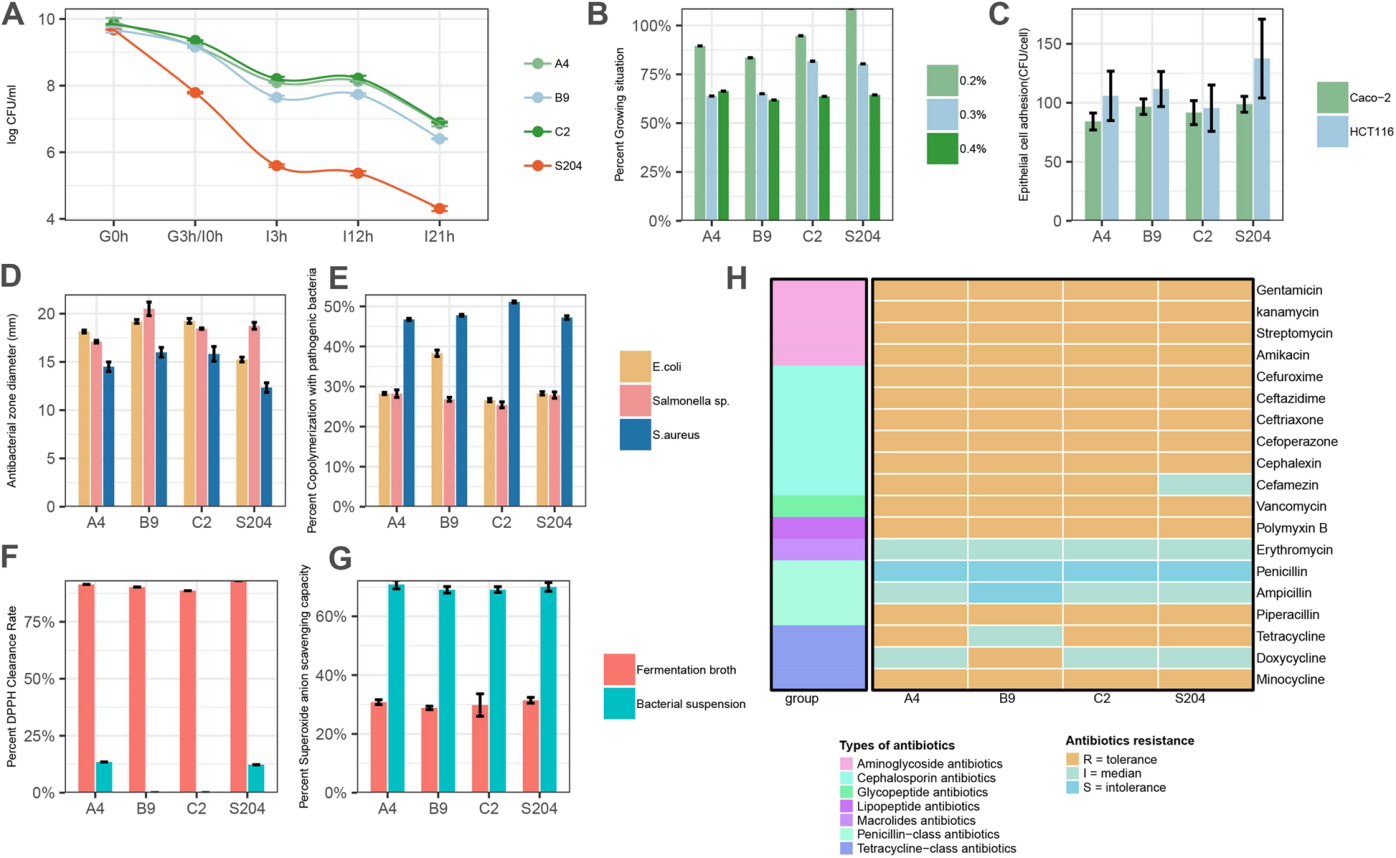
Screening and identification process of chicken source probiotics. **A** Evaluation of tolerance to different artificial gastric juices (G0h, G3h) and artificial intestinal juice (I0, I3h, I13h, I21h). **B** Bile salt rescreening results, percentage of survival at different bile salt concentrations, concerning the control group without bile salts. **C** Adhesion ability to different intestinal epithelial cells. **D** The inhibitory effect on three common pathogens. **E** Co-aggregation of the strains with pathogens. **F** Antioxidant activity by DPPH assay of supernatant and cells. **G** The antioxidant activity of supernatants and cells was measured by the removal rate of superoxide anion. **H** Heatmap of results for multiple antibiotics (R = tolerance, I = intermediary, S = intolerance)

We selected three strains with the greatest probiotic potential (A4, B3, and C2 all belonging to *P. acidilactici*) from a total of 829 strains and evaluated their probiotic properties. A laboratory-preserved strain of lactic acid bacteria, *P. acidilactici* S204 (CCTCC M2017002), was used as a control. This strain is a probiotic isolate previously obtained in our laboratory and is currently preserved as a patented strain at the CCTCC (China Center for Type Culture Collection). Since then, it has been successfully commercialized by domestic enterprises. The tolerance of probiotic strains to artificial gastric and intestinal fluid is a key indicator of their potential. Compared with S204, strains A4, B9, and C2 exhibited strong resistance to digestive fluids (Fig. 8A). The concentration of bile salts in the small intestine typically ranges from 0.03 to 0.3% [37]. These three strains showed strong tolerance to bile salts, with minimal impact on growth observed at 0.2% bile salt concentration. Moreover, more than 50% growth was achieved in the absence of bile salts even at a concentration of 0.4% (Fig. 8B).

Adhesion to the intestinal tract is essential for probiotic colonization and exerts beneficial effects. In this study, we evaluated the adhesion ability of the strains using two common human colon cancer cell lines, HT-29 and HCT116. The adhesion of strains A4, B9, and C2 to Caco-2 cells ranged from 84 to 98 CFU/cell, whereas their adhesion to HCT116 cells ranged from 96 CFU/cell to 112 CFU/cell (Fig. 8C). To assess the inhibitory capabilities against common pathogenic bacteria, we conducted inhibition zone assays and co-aggregation experiments. In the inhibition zone assays, B9 and C2 exhibited stronger inhibitory activities, with inhibition zones greater than 15 mm against all three pathogenic bacteria (Fig. 8D). Although A4 showed slightly weaker inhibitory activity, it still formed inhibition zones. In co-aggregation experiments with pathogens, the four strains showed a co-aggregation rate of approximately 28% with *Escherichia coli* and *Salmonella* sp. [38], while demonstrating a high aggregation ability of approximately 45% with *Staphylococcus aureus*, indicating their capacity to aggregate with pathogenic bacteria (Fig. 8E).

The antioxidant capacity is an important probiotic characteristic [39]. We investigated the ability of these four strains to scavenge DPPH radicals, hydroxyl radicals, and superoxide anions. The supernatant of all four strains exhibited extremely strong scavenging ability against DPPH radicals (approximately 90%), whereas the cell biomass showed weaker scavenging ability (approximately 13%; Fig. 8F). However, in the scavenging experiment of superoxide anions, the cell biomass of the four strains showed a clearance rate of 70%, whereas the supernatant had a clearance rate of only 30% (Fig. 7G).

The transfer of multiple antibiotic resistance genes to intestinal microbiota through plasmids has been previously reported [39]. Therefore, antibiotic resistance is used as the criterion to evaluate the safety of bacterial strains. These four strains were resistant to aminoglycoside antibiotics, cephalosporin antibiotics, glycopeptide antibiotics, and lipopeptide antibiotics (Fig. 8H). These strains showed intermediate resistance to macrolide antibiotics. Among the penicillin-class antibiotics, piperacillin and ampicillin exerted inhibitory effects on the four strains, with piperacillin showing the strongest inhibition. The four strains exhibited varying degrees of resistance and intermediate susceptibility to tetracycline-class antibiotics.

## Discussion

In the past decade, extensive research on the microbiome has highlighted the importance of the gut microbiota for host health. Changes in gut microbiota directly affect host health and disease. With the increasing availability of microbiome data, the construction of a comprehensive gene catalog for gut microbiota and systematic exploration of microbial community distribution hold great promise for future research across various species, including goats, horses, and chickens [1, 26, 40]. Chickens, a vital poultry species, have been used to the develop gut microbiome genomic and resistance gene databases for their intestinal microbiota. However, the composition of the gut microbiota in chickens can vary significantly depending on the species and farming practices. Currently, data on the gut microbiota of free-range indigenous chicken breeds are lacking.

In this study, we systematically investigated the gut microbiota of Jianghan chickens, an indigenous free-range breed. We analyzed the composition of gut microbial communities and provided comprehensive functional annotations. Our findings suggest that the gut microbiota of Jianghan chickens is similar to that of industrially raised chickens, with some difference. At the phylum level, Bacteroidetes and Firmicutes were the dominant bacteria in Jianghan chickens in Hubei, consistent with previous studies on chickens in chicken farms [23, 41, 42]. However, Bacteroidetes accounted for more than twice the relative abundance of Firmicutes. The Firmicutes/Bacteroidetes ratio, which is positively correlated with feed conversion ratio [43, 44], was lower in native chickens, possibly because they have a diverse diet that includes a large number of vegetable leaves, which differs from that of industrially raised broilers. Members of Bacteroidetes and Firmicutes can produce short-chain fatty acids in the intestine, which are commonly associated with obesity in humans [45], leading to a higher fat content and better edible flavor in Jianghan chickens. At the genus level, the most dominant microorganisms in Jianghan chickens in Hubei were *Phocaeicola* (16.3%) and *Bacteroides* (16.2%), which were similar to the gut microbial composition of chickens from chicken farm, but with different percentages [46–48]. The content of *Phocaeicola* and *Bacteroides* in the gut microorganisms of Jianghan chickens in Hubei was nearly 32.5%, which was almost twice that of yellow-finned chickens in the farm studied by Xu et al. [47].

Recent studies have emphasized the crucial role of the gut microbiome in host health and disease [49, 50]. However, most gut microbes cannot be cultured in the laboratory, which limits our understanding of their functions, including those related to substance metabolism and antibiotic resistance [26]. Metagenomic sequencing technology can be used to effectively study the functional gene composition of microbial communities. The annotation results in the KEGG database showed that the main function of the chicken intestinal microflora is metabolism. Among all the genes related to metabolism, the highest percentage was related to carbohydrate and amino acid metabolism. Amino acid metabolism breaks down proteins into peptides and amino acids [51]. Carbohydrates are the main source of nutrition for Jianghan chickens, and carbohydrate utilization is essential for chicken growth. Therefore, many genes related to carbohydrate metabolism function in chicken gut microbes would benefit chicken growth. Based on the annotation results of the CAZy database, it was found that the carbohydrate metabolism gene functions of the chicken gut microbial community were dominated by the synthesis of GHs and GTs, and there were also some genes whose functions were to provide CBMs and assist in the catabolism of PLs. This could be because the daily diet composition of the chickens sampled in this study different from that of industrially raised chickens.

The carrying of resistance genes by poultry intestinal microorganisms has attracted considerable attention [52, 53]. In the present study, we investigate the prevalence and diversity of ARGs in the gut microbiota of Jianghan chickens that did not receive antibiotic treatment. Surprisingly, despite the absence of antibiotic treatment, we observed persistence of multiple ARGs. Using the CARD, we identified the tet family of ARGs (tetW, tetO, tet40, tetX, tet37, tet44, tet32, and tet (W/N/W)) as the most abundant in the chicken gut, with tetQ, which confers resistance to tetracyclines, being the most prevalent. Tetracyclines have been widely used in animal feed for disease control and growth promotion, thus, their long-term use has likely contributed to the dissemination and persistence of tet genes in the gut microbiota of chickens. Several studies have reported the persistence of ARGs in microbial communities despite the ban of antibiotics as growth promoters in China in 2020 [54]. Moreover, Rovira et al. found that cessation of tetracycline use in farms did not result in an automatic reduction in resistance [9]. Research on chicken antibiotic resistance genes shows that *Escherichia*, *Enterococcus*, *Staphylococcus*, *Klebsiella*, and *Lactobacillus* were the major hosts of ARGs in the chicken gut microbiota. In particularly, *Lactobacillus*, a probiotic microbe commonly used in agricultural production, carried multiple ARGs and was positively correlated with the abundance of ISLhe63, indicating its potential risk in promoting antibiotic resistance in agricultural environments [54]. However, in our study, the main host of resistance gene in Jianghan chicken was different from the previous research conclusion. Based on the microbial composition analysis, we identified Bacteroidota and Bacillota were the major hosts of ARGs in the chicken gut microbiota. This conclusion is different from existing research and expands our understanding of resistance genes in free-range chickens, and main hosts of ARG are *Bacteroides* and *Alistipes* in Jianghan chicken at the genus level.

Microbial communities are known to be influenced by geographic characteristics, which can affect their diversity and function [55]. Local microbial communities are mainly shaped by pH, precipitation, and nutrients on a large scale [56], and climate change can significantly alter the complexity and diversity of local microbial communities [55]. In this study, we systematically investigated geographical differences in intestinal flora by sampling Jianghan chickens from four municipalities near the Yangtze River basin in Hubei Province, China. We observed significant differences in the bacterial community composition and diversity among the four samples, with the sample from JZ being notably distinct from the other three groups. We also found some differences in the fungal community composition among the samples from the four regions, although the general composition was similar. To investigate the differences in microbial composition among the four regions, we identified all the marker species (biomarkers) from the JZ samples, which were mainly from Firmicutes, Clostridia, Eubacteriales, Lachnospiraceae, and Oscillospiraceae branches. Several genera were identified in Osci llospiraceae. Differences in microbial composition differences can have an impact on the function of microbial communities [57]. In addition, we used metagenomic sequencing data to compare functional composition of the samples in the KEGG database. Using LEfSe analysis, we found that nearly all of the marker genes (biomarkers) in the functional genes were from JZ samples, with marker genes for the K00620 pathway and K00058 and K01997. Functional differences were observed in amino acid and carbohydrate metabolism (K00058), cellular processes (K01997), and environmental information processing (K01997). The results of species and functional difference analysis showed that there were certain differences between JZ samples and other samples, which provided guidance information for the subsequent probiotic screening work in this study.

Guided by the research results of the metagenome, 829 cultivable strains were isolated from the intestinal contents of Jianghan chickens. Several key factors must be considered when selecting a probiotic. Acid and bile resistance are of primary importance. Subsequently, their antioxidant capabilities, capacity to neutralize free radicals, and adherence to intestinal cells were assessed. Finally, the sensitivity of these strains to a range of antibiotics was examined. Among these criteria, acid and bile resistance tests can eliminate most strains. However, the results off the first screening are not always reliable. Therefore, this study conducted three successive large-scale acid and bile resistance screening experiments, which resulted in the identification of several strains with stable acid and bile resistance. In this study, a high-performance lactic acid bacterium strain, designated as *P. acidilactici* S204 (CCTTCC M2017002), was used as the control strain. The experimental findings demonstrate the excellent probiotic potential of three strains of *P. acidilactici* isolated from the Jianghan chicken gut. This substantiates the previously posited hypothesis that the Jianghan chicken gut hosts a substantial reserve of probiotic resources. Interestingly, in this study, the three selected strains of *P. acidilactici* that demonstrated resistance to gastric acid and bile salts, strong growth capability, inhibitory activity against pathogenic bacteria, and antioxidant capacity originated from the JZ sample. This raises the question of whether there is a connection between the unique characteristics of the JZ sample observed in sequencing studies and the selection of these beneficial strains. This potential connection may provide valuable guidance for future screening of probiotics in complex environments. In summary, this study began with metagenomic sequencing and revealed the basic species and functional protein composition of the gut microorganisms of native Hubei chickens, which guided subsequent strain screening work. The sequencing and microbial isolation results validated each other, deepening the feasibility of the conclusion and providing a large number of sequencing analysis results and microbial resources for the study of intestinal microorganisms in native Hubei chickens. The conclusion of this study is a good supplement to the study of the chicken intestinal microbiome and can be used as a control for the study of feed-raised chickens. Furthermore, study provides a systematic approach for the screening of probiotics from the gastrointestinal tracts of high-quality animal breeds. This approach, known as the top-down research model, initially employs omics technology to identify unique sample groups, followed by the selection of probiotics from these groups.

## Conclusion

Chickens are essential poultry species and a significant source of meat and eggs in human society. There are various chicken breeds in China, with white-feathered chickens being the primary meat-producers and green-shelled egg chickens used for egg production. With improvement in living standards, the demand for high-quality chicken meat and its by-products has increased. Jianghan free-range chickens, as a type of locally raised chicken, have gained popularity among consumers.

In this study, we found that the microbial composition of Jianghan chicken gut microbiota was similar to that of intensively raised white-feathered chickens at the species level. However, there were significant differences in the relative abundance of the predominant microbial taxa. Functional analysis revealed that carbohydrate, amino acid, and ester metabolism were the dominant functions observed in the gut microbiota of Jianghan chickens, they provide a variety of metabolic functions to the host. Surprisingly, despite the absence of antibiotic supplementation in the feed of these free-range chickens, a substantial number of resistance genes were detected in their gut microbiota, similar to the findings in intensively raised yellow-feathered chickens, with tetracycline resistance genes being the most prevalent. And we identified Bacteroidota and Bacillota were the major ARG hosts at the phylum level, *Bacteroides* and *Alistipes* are the main ARG hosts at the genus level. Furthermore, from a screening of nearly a thousand bacterial strains, we identified three strains of *P. acidilactici* as potential probiotics, exhibiting robust tolerance and demonstrating antimicrobial and antioxidative properties. These findings provide insights into the composition of gut microbiota of Jianghan chickens and highlight the presence of potential probiotic strains that may contribute to their unique characteristics and offer benefits to the host.

This study has certain limitations. For instance, the sample size can be further increased. The screened *P. acidilactici* with probiotic potential has not yet undergone animal feeding experiments. These limitations should be addressed in future studies.

## Methods

### Sample collection and strain isolation

In this study, a total of 34 intestinal and content samples were collected from Jianghan chickens in four different regions of Hubei Province, China. The Jianghan chicken is a unique breed with a history of three centuries in the Jianghan region of China. It is a lightweight breed with a tendency to startle easily and fly at higher altitudes, featuring low feeding requirements and a slow growth rate. Due to its strong stress reaction, intensive farming is unsuitable, and the predominant farming method involves free-ranging on flat land. Specifically, samples were collected from chickens raised in Jingzhou (*n* = 8), Yichang (*n* = 10), Huanggang (*n* = 8), and Huangshi (*n* = 8). The chickens are randomly selected from flocks raised by local farmers and are fed corn as the primary ingredient, supplemented with vegetable leaves. All experimental chicken samples were Jianghan chickens, aged 10 to 12 months, and all were healthy and energetic hens. Sample collection will be completed between April 2022 and May 2022. This study has an ethical clearance number of HZAUCH-2023–0013. After the test chickens were bled and euthanized, their body surface was disinfected with 75% ethanol. The cecum tissue was quickly dissected, and the ends were tied tightly using sterile cotton thread. A portion of the removed intestinal tissue and anaerobic gas-producing bag were transferred to an anaerobic sealed bag, sealed, and briefly stored at 4 ℃ for microbial isolation. The rest of the samples were frozen in liquid nitrogen, transported to the laboratory, and stored in a freezer at − 80 ℃ for DNA extraction.

To isolate the bacterial strains, 5 g of each cecum sample was weighed in a centrifuge tube and incubated with 45 ml of sterile water for 20 min at 37 ℃ in a shaker at 200 r/min. The supernatant was then diluted in a gradient, and dilutions of 10^5^, 10^6^, and 10^7^ were applied to MRS and LBS media, respectively. The media were incubated in an anaerobic incubator at 37 ℃ and a 37 ℃ incubator for 24 h. The colonies near the yellowing colonies on the media were picked, and single colonies near the picked colonies were isolated by multiple streaking until purified. The 16S rDNA sequences were sequenced by Sanger sequencing, and the sequencing results were compared to confirm the identity of the bacterial strains before they were conserved.

### DNA extraction, library preparation, and sequencing

The microbial genomic DNA samples were extracted according to the manufacturer’s Magnetic Soil And Stool DNA Kit (TINGGEN) and stored at − 80 ℃. To ensure the purity and quality of the extracted DNA, a NanoPhotometer and a Qubit 3.0 were used, respectively. The integrity of the DNA was assessed by agarose electrophoresis.

To prepare the DNA for sequencing, 0.5 μg genomic DNA was randomly fragmented using Bioruptor Pico and then filtered with magnetic beads. The Adaptor was added and the DNA was repaired, followed by magnetic bead purification. PCR was performed to amplify and enrich the products. The double-stranded PCR library was then purified to unchain and loop to form a single-stranded circular DNA. Rolling ring amplification (RCA) technology was used to form the DNA nanosphere (DNB), which was loaded into the chip and fixed through a fully automatic sample loading system.

After library construction, the sequencing library was sequenced on DNBSEQ-T7 at Bioyi Biotechnology Co., Ltd. located in Wuhan, China.

### Metagenomic data analysis methods

#### Quality control

Samples are sequenced on the platform to get image files, which are transformed by the software of the sequencing platform, and the original data in FASTQ format (Raw Data) is generated. Sequencing data contains a number of connectors, low-quality Reads, so we use fastp (v0.21.0) software to filter the sequencing data to get high-quality sequence (Clean Data) for further analysis.

#### Analysis of species diversity based on reads

Clean Data was used for species annotation by using kraken2. After that, bracken and KrakenTools were used for statistics and format conversion of the results. Species distribution results were presented using krona.

#### Genome assembly and the construction of non-redundant gene sets

1. Genome assembly: the data after quality control are assembled by megahit or spades, and biotool is used to rank and count the genomes. The contig of genes were annotated using kraken2, with non-microbial data removed to obtain microbial genomes.
2. Construction of non-redundant gene sets: MetaGeneMark was used to predict the genes, and build non-redundant gene sets and protein sets by CD-hit.
3. Functional annotation: the protein set was annotated with Interproscan to extract the annotation information of TIGRFAMs, Pfam and GO databases, using diamond to align the protein set to KEGG, NR, SwissProt, and COG databases, retaining the best alignment coverage above 30% as the annotation result.

#### CAZy, CARD, and VFDB database annotations

After the extraction of the genomic-encoded proteins, the encoded proteins were aligned to the database with blastp, and the best result with an alignment of coverage greater than 30% was retained as the CAZy annotation result. CARD and VFDB annotation analysis was performed using abricate (v1.0.1), retaining the best results of the alignment of coverage greater than 50% and identity greater than 75% as the annotation results.

### Tolerance test of artificial gastrointestinal juice

#### Preparation of bacterial suspension

The selected strains were inoculated at 1% (v/v) into MRS liquid medium and incubated at 37 ℃ for 24 h. After activation, the third-generation bacterial suspension was collected by centrifugation at 4000*g* for 10 min. The bacterial cells were washed twice with sterilized PBS and resuspended in 10 mL of PBS to prepare a uniform bacterial suspension.

#### Artificial simulated gastric fluid

PBS buffer solution was prepared and adjusted to pH 2.5 with 1 mol/L HCl. Then, 0.3% pepsin was added and completely dissolved. The solution was filtered through a 0.22-μm microporous membrane for sterilization and kept for later use.

#### Artificial simulated intestinal fluid

PBS buffer solution was prepared and supplemented with 10 g/L pancreatin and 3 g/L Cow bile powder. The pH was adjusted to 8.0 with 0.1 mol/L NaOH, and the solution was fully dissolved. After filtration through a 0.22-μm microporous membrane for sterilization, it was set aside for later use.

For the tolerance test, 1 mL of bacterial suspension was inoculated into 9 mL of pH 2.5 sterilized artificial gastric fluid. After thorough mixing, it was incubated at 37 ℃ for 3 h, and the viable cell count was determined at 0 and 3 h. Then, 1 mL of the sterile gastric fluid treated for 3 h was inoculated into 9 mL of pH 8.0 sterilized artificial intestinal fluid. After thorough mixing, it was incubated at 37 ℃ in a constant-temperature incubator. The viable cell count was determined at 0, 3, 9, and 21 h, and the survival rate was calculated.

#### Bile salt tolerance screening of lactic acid bacteria

The strains at the end of the cultivation cycle were inoculated at 2% into MRS liquid medium containing 0.2 and 0.3% bile salts, with MRS liquid medium without ox bile powder as a blank control. The cultures were incubated at 37 ℃ with samples taken hourly to measure the absorbance value at 620 nm. The cultivation was terminated when this absorbance value increased by more than 0.3 units. The bile salt tolerance was evaluated based on the length of the lag phase, which refers to the time difference required for the experimental group and the blank group strains to increase the absorbance by 0.3 units.

#### Measurement of antioxidant capacity

Due to the variation between strains, there are differences in the distribution of antioxidant substances. The fermentation supernatant and cell suspension of the selected and control strains were used for the measurement of antioxidant capacity.

#### Preparation of cell suspension

The bacterial suspension was activated and centrifuged at 4 ℃ and 4000*g* for 10 min. The fermentation supernatant was collected and stored, and the bacterial cells were washed three times with sterilized PBS. After resuspension, the bacterial suspension was adjusted to a concentration of 10^8^ CFU/mL (OD_600_ = 1.0).

#### Determination of DPPH radical scavenging capacity

A total of 1 mL fermentation supernatant (cell suspension) was added to 1 mL of DPPH ethanol solution (0.2 mmol/L). After thorough mixing, it was placed at room temperature under dark condition for 30 min. Then, it was centrifuged at 4 ℃ and 6000*g* for 10 min, and then the supernatant was used to measure the absorbance (*A*_*i*_) at 517 nm. The blank group (*A*_*j*_) is prepared by replacing the DPPH anhydrous ethanol solution with an equal volume of anhydrous ethanol. The control group (*A*_0_) is prepared by replacing the sample solution with an equal volume of distilled water. Blank zeroing is performed using a mixture of equal volumes of distilled water and anhydrous ethanol. DPPH radical scavenging capacity (%) = [1 − (*A*_*i*_ − *A*_*j*_)/*A*_0_] × 100%

### Statistical analysis

Data manipulation and visualization were performed through the R meta package tidyverse (1.3.0) [58]. *T*-test, Kruskal–Wallis rank sum test, and Wilcoxon rank sum test were performed through functions “t.test,” “kruskal.test,” and “wilcox.test” in package “stats” (4.2.1). Before analysis, the samples were rarefied to uniform depth based on the lowest sample sequence to eliminate the influence of different sequencing depths. Alpha diversity indices (Shannon, pielou’s eveness, observed species, and faith’s pd) were calculated using package ‘vegan’ [59]. Beta diversity metrics (Jaccard dissimilarity, Bray–Curtis dissimilarity) as well as PCA were conducted using function “rda” in vegan (2.7) followed by an ADNOIS test to measure the changes related to sampling sites [60]. LEfSe is an algorithm that can identify high-dimensional biomarkers (genes, pathways, or taxa) and characterize the differences between two or more biological conditions. We use local software to do the LEfSe analysis. IQ-tree was used to construct the phylogenetic tree, and the tree-building mode of automatically matching the best model was used [61]. All visualizations are done in R, mainly based on ggplot2.

## Data Availability

All data generated or analyzed during this study are included in this published article and its Additional file. All of the original sequences obtained in this work have been deposited in the National Center for Biotechnology Information (NCBI) under project number PRJNA1031251.

## Abbreviations

JZ: Jingzhou City, Hubei Province, China
YC: Yichang City, Hubei Province, China
HS: Huangshi City, Hubei Province, China
HG: Huanggang City, Hubei Province, China
PCA: Principal component analysis
LEfSe: Linear discriminant analysis Effect Size
ARGs: Antibiotic resistance genes
